# Relationship of anthropometric indices with rate pressure product, pulse pressure and mean arterial pressure among secondary adolescents of 12–17 years

**DOI:** 10.1186/s13104-021-05515-w

**Published:** 2021-03-17

**Authors:** Godfrey Katamba, Abdul Musasizi, Mivule Abdul Kinene, Agnes Namaganda, Francis Muzaale

**Affiliations:** 1 Department of Physiology, College of Health, Medicine and Life Sciences, King Ceasor University, Kampala, Uganda; 2 Department of Biochemistry, College of Health, Medicine and Life Sciences, King Ceasor University, Kampala, Uganda; 3 Department of Anatomy, College of Health, Medicine and Life Sciences, King Ceasor University, Kampala, Uganda; 4 Department of Physiology, Faculty of Health Sciences, Busitema University, Mbale, Uganda; 5 Department of Physiology, Faculty of Health Sciences, Gulu University, Gulu, Uganda

**Keywords:** Anthropometric indices, Rate pressure product, Pulse pressure, Mean arterial pressure

## Abstract

**Objectives:**

To determine the correlation between anthropometric indices and the selected hemodynamic parameters among secondary adolescents aged 12–17 years.

**Results:**

Our findings showed weak positive correlation between generally body surface area, neck circumference and conicity index with the hemodynamic parameters (systolic blood pressure, diastolic blood pressure, resting pulse rate, mean arterial pressure, rate pressure product and pulse pressure). However, the ponderosity index, body mass index and waist hip ratio showed negative weak correlations with the hemodynamic parameters. There was a significant difference in pulse pressure among the BMI categories. All parameters showed significant (p < 0.05) differences across the categories of neck circumference and waist hip ratio. Generally, in multivariate regression analysis, anthropometric indices showed significant prediction of the hemodynamic parameters.

**Supplementary Information:**

The online version contains supplementary material available at 10.1186/s13104-021-05515-w.

## Introduction

The role of anthropometric measurements in estimation of adiposity is widely used in both research and clinical settings. The indices, such as; bod mas index (BMI), and the waist hip ratio (WHR) have been used commonly to define overweight and obesity status, across various age categories. Recently, more indices such as the conicity index (CI); ponderosity index (PI); body surface are (BSA), and neck circumference (NC), attracted great attention. Overweight and obesity is highly associated with a number of non-communicable diseases including cardiovascular disease, diabetes mellitus, cancers, arthritis, ovarian dysfunction and so forth. Globally, based on the world health organization (WHO), the prevalence of obesity has tripled since 1975, as in 2016 only more than 1.9 billion adults of age 18 years and greater were overweight, of which 650 million were obese [1]. Obesity plays a very vital role in the development of cardiovascular disease (CVD) [2–4]. Several simple anthropometric parameters have been greatly associated with cardiovascular risk factors [5] and thus their relationship with hemodynamic parameters needs to be explored. The rate pressure product (RPP) also known as the cardiovascular product, is the measure of stress put on the cardiac muscle, based on the number or times it needs to contract per minute and the arterial blood pressure that is pumping against it [6]. It is a direct indication of the energy demand of the heart and thus good measure of energy consumption by the heart. Anthropometric indices such as WHR, waist circumference (WC), and BMI were found to be significantly correlated with RPP among health young adults [7, 8], and an important predictor of cardiovascular events [9]. The mean arterial pressure (MAP) is physiologically the average pressure in the arteries during a single cardiac cycle [10] and it is useful indicator of the pressure necessary for the adequate perfusion to vital organs. Additionally, the pulse pressure (PP) is an indirect marker of arterial stiffness and distensibility [11]. Both MAP and PP are important indicators of the pressure inside arteries and the extent of vasoconstriction or dilation. These have been found to be associated with anthropometric indices in elderly populations [12] and young adults elsewhere [13]. Further exploration of this relationship is needed for secondary school adolescents in Ugandan settings for a reliable comparison and better understanding of the cardiovascular related pathophysiology.

## Main text

### Study design and data

Data included in this study was part of a larger cross-sectional study which included up to 616 [14] adolescents aged 12–19 years in Mbarara municipality, southwestern Uganda. In this paper, only data of 485 participants aged 12–17 years was analyzed.

### Anthropometric measurements

The methods were already described in detail by Katamba et al. [15]. Briefly, height (Ht) was measured using a wall mount height board without shoes in centimetres (cm) [2]. Weight (Wt) was measured to the nearest 0.5 kg using a standard weighing scale (Seca 762, GmbH & Co. KG, Hamburg, Germany) and participants were encouraged to put on light clothing with no items in the pockets and shoeless [3]. Waist circumference (WC) was measured by an inelastic flexible measuring tape with the participant standing while hip circumference (HC) at the level of the greater trochanter to the nearest 0.1 cm using an inelastic flexible measuring tape with the participant standing. Neck circumference (NC) was used as a surrogate measure for upper body adipose tissue distribution, measured at the level of the laryngeal prominence using an inelastic flexible measuring tape.

## Computations

$$ {\text{Ponderosity\,index }}\left( {{\text{PI}}} \right) = \frac{{Weight\,in\,kg}}{{Height\,in\,cubic\,meters}}{ }in\frac{{{\text{kg\,}}}}{{{\text{m}}^{3} }} $$

[16]

$$ {\text{Body\,surface\,area }}\left( {{\text{BSA}}} \right) = \sqrt {\frac{{Height\,\left( {cm} \right)\,x\,{ }weight{ }\left( {kg} \right)}}{3600}{ }} in\;{\text{m}}^{2} $$

based on the Mosteller formula [17]

$$ {\text{ Conicity\,index }}\left( {{\text{CI}}} \right) = \frac{{waist\,circumference\,\left( m \right)}}{{0.109\sqrt {\frac{{weight\,\left( {kg} \right)}}{{height\,\left( m \right)}}} }}{\text{in\,kgm}}^{ - 3} . $$

[18]

### Blood pressure and heart rate

Blood pressure was measured using a digital blood pressure machine (Scian SP-582 Digital BP Monitor, Honsun, Jiangsu, China (Mainland) as already described by Katamba et al. [15]. Each participant was allowed to rest quietly for 5 min, sitting on a chair with the back supported and feet on the floor [14]. The participant was asked to remain calm and quiet as the machine begun to measure automatically after pressing the start button. When the reading was complete, the monitor displayed the BP and the resting pulse rate on the digital panel [19]. Three readings were recorded per participant at 5 min’ interval as recommend by the WHO steps surveillance guidelines for non-communicable diseases [20]. The average of the 2nd and 3rd respective BP measurements was used as the subject’s BP, respectively. The rate pressure product (RPP) was computed as the product of systolic blood pressure and the resting pulse rate (RPP = SBP x RPR). Pulse pressure was the difference between systolic and diastolic blood pressure (PP = SBP − DBP) while Mean arterial pressure (MAP = DBP + 1/3 PP).

### Statistical analysis

The subjects were classified into different groups using anthropometric indices such as BMI, NC and WHR. The WHO 2007 BMI categories for age were used to classify adolescents into obesity categories [21]. Based on WHR, the subjects were classified into two groups, i.e., WHR < 0.90 and WHR ≥ 0.90 (WHO cutoff points). The NC cut-offs for boys and girls were 30.75 cm and 29.75 cm, respectively [22]. To compare two samples of a continuous variable, an unpaired t-test was used whereas for those with three categories, one‑way ANOVA test was implemented. The anthropometric indices were tested for normality using the skewness and kurtosis test. The Pearson moment correlation coefficient was used to determine the correlations between anthropometric indices and the hemodynamic variables parameters followed by linear regression analysis. The anthropometric indices that showed a high variance inflation factor were removed from the final regression model to control for collinearity. *A p* value < 0.05 was considered to be statistically significant. The analysis of data was done by Stata software version 13.0 (College Station, Texas, USA).

## Results

### Physical characteristics of study participants by sex

The mean age of the participants was 14.9 ± 1.6 years, the SBP and DBP of the participants were 111.9 ± 8.9 mmHg which differed significantly by sex and 65.0 ± 7.4 mmHg, respectively. The mean resting pulse rate (RPR) was 75.0 ± 8.1 bpm. The mean RPP was 84.1 ± 12.8 × 10^2^ mmHgbpm, while the mean PP was 46.9 ± 7.9 mmHg as showed in Table 1. A total of 6.6% (n = 32) of the participants was obese based on the WHO 2007 BMI for age categories.

**Table 1:** showing physical characteristics of the participants by compared by sex

Variable	Male (n = 173)	Female (n = 312)	p-value	Total (n = 485)
	Mean ± SD	Mean ± SD		Mean ± SD
Age (years) Ht (cm) Wt (kg) WC (cm) NC (cm) HC (cm) BMI (kgm^−2^) WHR PI (kgm^−3^) BSA (m^2^) CI (kgm^−3^) RPR (bpm) SBP (mmHg) DBP (mmHg) RPP × 10^2^ (mmHgbpm) MAP (mmHg) PP (mmHg)	14.5 ± 1.5 157.2 ± 7.8 55.0 ± 7.3 67.6 ± 6.3 29.7 ± 2.5 79.8 ± 8.4 22.3 ± 2.9 0.85 ± 0.06 14.2 ± 2.2 1.55 ± 0.12 1.05 ± 0.09 73.6 ± 7.5 109.6 ± 10.9 64.3 ± 6.9 80.8 ± 11.9 79.4 ± 7.5 45.3 ± 8.6	15.1 ± 1.5 156.9 ± 7.4 60.2 ± 7.6 69.9 ± 6.9 29.8 ± 1.6 93.3 ± 11.1 24.5 ± 2.3 0.75 ± 0.06 15.6 ± 1.8 1.6 ± 0.13 1.04 ± 0.08 75.8 ± 8.4 113.2 ± 7.3 65.4 ± 7.6 86.0 ± 12.9 81.3 ± 6.6 47.8 ± 7.4	< 0.01 0.620 < 0.01 < 0.01 0.368 < 0.01 < 0.01 < 0.01 < 0.01 < 0.01 0.072 0.005 < 0.01 0.123 < 0.01 < 0.01 < 0.01	14.9 ± 1.6 156.9 ± 7.6 58.3 ± 7.9 69.1 ± 6.8 29.8 ± 1.9 88.5 ± 12.1 23.7 ± 2.8 0.79 ± 0.08 15.1 ± 2.1 1.6 ± 0.13 1.04 ± 0.08 75.0 ± 8.1 111.9 ± 8.9 65.0 ± 7.4 84.1 ± 12.8 80.6 ± 6.9 46.9 ± 7.9

*SD* standard deviation

### Comparison of hemodynamic variables based on BMI categories

Additional file 1: Table S1 shows the comparison hemodynamic parameters across the BMI categories. Data analysis was done by one‑way ANOVA. There was a significant difference in the cardiovascular parameters across ss all the BMI categories.

### Comparison of hemodynamic variables-based on neck circumference and waist hip ratio

Additional file 1: Table S2 depicts the effect of NC and WHR on hemodynamic parameters. Comparison analysis was done using the unpaired *t*‑test. The subjects of normal NC cm showed significant difference in all hemodynamic parameters with those of high NC (p < 0.01). The same significant differences were found between the WHR categories.

### Correlation analysis

Correlation analysis of hemodynamic parameters with anthropometric indices was done as shown in Additional file 1: Table S3. Significant positive correlation coefficients were found between body surface area and neck circumference with RPP, MAP and PP. However, some negative coefficients were found for WHR and PI.

### Step wise regression analysis

All anthropometric indices as shown in Table 2 were significantly associated with the cardiovascular parameters (RPP, MAP and PP) and this was controlled for sex. The multiple and stepwise regression analysis for of anthropometric indices in relation to hemodynamic parameters is shown in Table 3. BMI and WHR controlled for sex significantly predicted the rate pressure product (RPP) with explained variability of 7%. This prediction improved after addition of conicity index in step 2 (R^2^ = 0.149). The explained variability in RPP later improved (R^2^ = 0.149 vs 0.169) after addition of the ponderosity index in step 3 of the analysis. The same improvement in the explained variability were observed for MAP and PP.

**Table 2:** Bivariate linear regression analysis of anthropometric indices with the cardiovascular parameters adjusted for sex

Variables	Rate pressure product			Mean arterial pressure			Pulse pressure		
	R^2^	SEE	F	R^2^	SEE	F	R^2^	SEE	F
BMI WHR CI PI BSA	0.052 0.057 0.097 0.077 0.066	22.2 928.9 3407.9 28.7 446.9	13.3* 14.7* 25.9* 20.0* 16.9*	0.021 0.056 0.043 0.047 0.091	0.1 5.1 19.2 0.2 2.4	5.1* 14.4* 10.8* 11.9* 24.2*	0.038 0.023 0.031 0.025 0.082	0.1 5.9 22.0 0.2 2.8	9.6* 5.8* 7.6* 6.2* 21.6*

*SEE* standard error of estimation, *R*^*2*^ coefficient of determination, *F* F-Statistic

^*^p < 0.05

**Table 3:** Stepwise analysis showing changes in multiple regression coefficient of determination (R^2^) with addition of different anthropometric indices controlled for sex

Stages of analysis	Rate pressure product			Mean arterial pressure			Pulse pressure		
	R^2^	SEE	F	R^2^	SEE	F	R^2^	SEE	F
^1^BMI and WHR ^2^BMI, WHR and CI ^3^BMI, WHR, CI and PI	0.072 0.149 0.169	21.9 26.9 59.1	12.4* 20.9* 19.5*	0.060 0.117 0.169	0.121 0.150 0.322	10.2* 15.9* 19.5*	0.038 0.038 0.084	0.139 0.178 0.386	6.4* 4.8* 8.8*

*SEE* standard error of estimation, *R*^*2*^ coefficient of determination, *F* F-Statistic

^*^p < 0.05

### Multiple linear regression equations for different stages of analysis

Mean arterial pressure (MAP);

$$ \begin{gathered}^{1} MAP \, = \, 102.4 - 0.17BMI \, {-} \, 22.8WHR, \hfill \\^{2} MAP \, = \, 75.3 \, {-} \, 0.35BMI \, + 136.8CI - 32.5WHR, \hfill \\^{3} MAP \, = \, 68.6 \, + \, 1.93BMI \, - 21.2WHR \, - \, 2.4PI + 108.6CI. \hfill \\ \end{gathered} $$

Pulse pressure (PP);

$$ \begin{gathered}^{1} PP = \, 34.9 \, + \, 0.38BMI \, + \, 0.26WHR, \hfill \\^{2} PP \, = \, 37.0 \, + \, 0.34BMI \, + \, WHR \, - 10.6CI, \hfill \\^{3} PP \, = \, 29.9 \, + \, 2.0BMI \, + \, 13.1WHR \, {-} \, 2.5PI \, {-} \, 4.0.8CI. \hfill \\ \end{gathered} $$

Rate pressure product (RPP);

$$ \begin{gathered}^{1} RPP \, = \, 11505.8 \, {-} \, 59.7BMI \, - \, 2910WHR, \hfill \\^{2} RPP = \, 5713.7 \, + \, 51.0 \, BMI \, - 4983WHR \, + \, 29172CI, \hfill \\^{3} RPP = \, 4944.5 \, + \, 232.5BMI \, {-} \, 3676.8 \, WHR \, - 272.8PI + 25919.2CI. \hfill \\ \end{gathered} $$

## Discussion

Our findings showed weak correlations between the anthropometric indices and the hemodynamic parameters. The correlations were all positive for NC and CI. However, the PI, BMI and WHR showed negative weak correlations with the hemodynamic parameters. In regression analysis, combination of all anthropometric indices significantly predicted the hemodynamic parameters among our adolescents with improved explained variability. These observations carry physiological significance, as the increase in anthropometric indices results into increased stress on the cardiovascular system, as indicated by increase in hemodynamic parameters. Rate pressure product as marker of myocardial stress through surrogate indication myocardial oxygen consumption (m*V*O_2_) increased with increase in the anthropometric indices, as was also shown by pulse pressure, mean arterial pressure and blood pressure. Our findings are in agreement with those from a study that involved 104 male young adults aged 20–25 years. The study found a positive correlation between anthropometric indices and rate pressure product [7]. Additionally, a study among primary pupils of 6–14 years found anthropometric indices such as height, weight, waist hip ratio to be significantly correlated with both blood pressure and pulse pressure [23]. The same findings were reported among secondary adolescents of 10–18 years in Gombe Nigeria [24]. Among the 397 apparently healthy men and women from Congolese in the south port city, waist circumference was found to be associated with pulse pressure and the strength of the associations varied with age, and blood pressure status [25]. In china, the simple anthropometric indices such as BMI, WC and WHR were found to be useful predictors for cardiovascular risk [5]. The same observations were reported among Brazilian adolescents [26] and in Korean women aged 40–69 years [27]. In the elderly population, it was found that higher values of anthropometric indices such as waist to height ratio and conicity index were closely related to increased diastolic BP, body fat and lipid profiles [28]. The same closely related findings were reported among the postmenopausal women in Tehran in Iran [29]. Neck circumference in children was found to show very comparable associations as those of BMI, WHR, WHtR and was closely associated cardiovascular disease risk factors among the 324 children aged 9–13 years in Greece [30].

## Conclusion

A positive relationship between the anthropometric indices and hemodynamic parameters was found in general. All anthropometric indices combined predict the hemodynamic parameters better than a single index of adiposity.

## Limitations

These include; (1) our results included only secondary school adolescents and hence may not be generalized across all adolescents in the general public, (2) the sample size was small to draw very valid conclusions and hence this calls for a larger national wide survey, (3) being a cross sectional study, no information on causality was obtained.

## Supplementary Information

**Additional file 1:** **Table S1**. Comparison of hemodynamic variables between Thinness, overweight and obese BMI subjects. **Table S2**. Comparison of hemodynamic variables based on neck circumference and waist hip ratio categories. **Table S3**. Correlation between anthropometric indices and hemodynamic parameters.

## Data Availability

The dataset is available on request from the corresponding author.

## Abbreviations

ANOVA: Analysis of variance
BMI: Body mass index
BP: Blood pressure
BSA: Body surface area
CI: Conicity index
DBP: Diastolic blood pressure
HC: Hip circumference
Ht: Height
MAP: Mean arterial pressure
NC: Neck circumference
PI: Ponderosity index
PP: Pulse pressure
RPP: Rate pressure product
RPR: Resting pulse rate
SBP: Systolic blood pressure
SD: Standard deviation
SE: Standard error
WC: Waist circumference
WHO: World health organization
WHR: Waist hip ratio
Wt: Weight
