# Supplementary material for: Relationship of anthropometric indices with rate pressure product, pulse pressure and mean arterial pressure among secondary adolescents of 12–17 years
Source: BMC Res Notes. 2021 Mar 17;14:101. doi: 10.1186/s13104-021-05515-w (PMC7968204; doi:10.1186/s13104-021-05515-w)
Supplement: Supplementary file 1 — Additional file 1: Table S1. Comparison of hemodynamic variables between Thinness, overweight and obese BMI subjects. Table S2. Comparison of hemodynamic variables based on neck circumference and waist hip ratio categories. Table S3. Correlation between anthropometric indices and hemodynamic parameters. [file 13104_2021_5515_MOESM1_ESM.docx]

Supplementary material 1 for **Relationship of anthropometric indices with rate pressure product, pulse pressure and mean arterial pressure among secondary adolescents of 12-17 years**

Table S1: Comparison of hemodynamic variables between Thinness, overweight and obese BMI subjects

| Variables | Thinness (185) | Overweight (n=268) | Obese (n=32) | p-value |
| --- | --- | --- | --- | --- |
| **DBP (mmHg)**  **SBP (mmHg)**  **RPR (mmHg)**  **RPPx10^2^ (mmHgbpm)**  **MAP (mmHg)**  **PP (mmHg)** | 67.0±8.7  112.0±10.6  77.2±10.0  86±15  82.0±8.2  44.9±9.5 | 63.9±6.1  112.4±6.7  73.9±6.4  83±10  80.1±5.7  48.4±5.8 | 62.1±5.6  107.9±12.9  71.9±5.1  78±13  77.4±7.0  45.8±10.9 | p<0.001  0.027  p<0.001  p<0.001  p<0.001  p<0.001 |

Data expressed in mean±SD form. Statistical test was one‑way ANOVA. n=Number of subjects, SD=Standard deviation

Table S2: Comparison of hemodynamic variables based on neck circumference and waist hip ratio categories

| Variables | Neck circumference (cm) | | | Waist hip ratio | | |
| --- | --- | --- | --- | --- | --- | --- |
|  | Normal (n=265) | High (n=220) | p | Normal (n=374) | High (n=111) | p |
| DBP (bpm)  SBP (bpm)  RPR (bpm)  RPPx10^2^ (mmHgbpm)  MAP (mmHg)  PP (mmHg) | 63.4±0.4  108.8±0.5  74.1±0.5  80.7 ±12.0  78.5 ±6.0  45.3 ± 7.6 | 66.9±0.5  115.7±0.6  76.1 ±0.5  88.2± 12.5  83.2 ±7.2  48.8 ±7.9 | <0.01  <0.01  <0.01  <0.01  <0.01  <0.01 | 65.6±0.4  112.9±0.5  75.7±0.4  85.6±13.1  81.4 ± 7.2  47.3 ±8.5 | 62.0±0.5  108±0.7  72.7±0.6  79.1± 10.0  78.1 ±5.5  45.8 ±5.6 | <0.01  <0.01  <0.01  <0.01  <0.01  0.041 |

Data expressed in mean±SD form. Statistical test was unpaired t-test, n=Number of subjects, SD=Standard deviation

Table S3: Correlation between anthropometric indices and hemodynamic parameters

| Variable | NC | BMI | WHR | PI | BSA | CI |
| --- | --- | --- | --- | --- | --- | --- |
| SBP (mmHg)  DBP (mmHg)  RPR bpm)  RPP (mmHgbpm)  MAP (mmHg)  PP (mmHg) | 0.44**  0.25**  0.11**  0.32**  0.36**  0.27** | 0.10*  -0.06  -0.14**  -0.03  -0.001  0.17* | -0.24**  -0.19**  -0.15**  -0.23**  -0.24**  -0.09* | -0.04  -0.14**  -0.16**  -0.12**  -0.12**  0.09* | 0.39**  0.18**  -0.01  0.21**  0.30**  0.27** | 0.25**  0.21**  0.22**  0.28**  0.26**  0.08 |

*p<0.05, **p<0.01; Independent variables: NC; neck circumference, BMI; Body mass index, WHR; Waist hip ratio, PI; ponderosity index, BSA; Body surface area, CI; conicity index.
